# Construction of liver hepatocellular carcinoma-specific lncRNA-miRNA-mRNA network based on bioinformatics analysis

**DOI:** 10.1371/journal.pone.0249881

**Published:** 2021-04-16

**Authors:** Ruifang Wang, Xiaobo Hu, Xiaorui Liu, Lu Bai, Junsheng Gu, Qinggang Li

**Affiliations:** 1 Department of Nuclear Medicine, The First Affiliated Hospital of Zhengzhou University, Zhengzhou, Henan, China; 2 Department of Infectious Diseases, The First Affiliated Hospital of Zhengzhou University, Zhengzhou, Henan, China; University of Calgary, CANADA

## Abstract

Liver hepatocellular carcinoma (LIHC) is one of the major causes of cancer-related death worldwide with increasing incidences, however there are very few studies about the underlying mechanisms and pathways in the development of LIHC. We obtained LIHC samples from The Cancer Genome Atlas (TCGA) to screen differentially expressed mRNAs, lncRNAs, miRNAs and driver mutations. The Kyoto Encyclopedia of Genes and Genomes (KEGG) pathway, Gene ontology enrichment analyses and protein–protein interaction (PPI) network were performed. Moreover, we constructed a competing endogenous lncRNAs-miRNAs-mRNAs network. Finally, cox proportional hazards regression analysis was used to identify important prognostic differentially expressed genes. Total of 1284 mRNAs, 123 lncRNAs, 47 miRNAs were identified within different tissues of LIHC patients. GO analysis indicated that upregulated and downregulated differentially expressed mRNAs (DEmRNAs) were mainly associated with cell division, DNA replication, mitotic sister chromatid segregation and complement activation respectively. Meanwhile, KEGG terms revealed that upregulated and downregulated DEmRNAs were primarily involved in DNA replication, Metabolic pathways, cell cycle and Metabolic pathways, chemical carcinogenesis, retinol metabolism pathway respectively. Among the DERNAs, 542 lncRNAs-miRNAs-mRNAs pairs were predicted to construct a ceRNA regulatory network including 35 DElncRNAs, 26 DEmiRNAs and 112 DEmRNAs. In the Kaplan‐Meier analysis, total of 43 mRNAs, 14 lncRNAs and 3 miRNAs were screened out to be significantly correlated with overall survival of LIHC. The mutation signatures were analyzed and its correlation with immune infiltrates were evaluated using the TIMER in LIHC. Among the mutation genes, TTN mutation is often associated with poor immune infiltration and a worse prognosis in LIHC. This work conducted a novel lncRNAs-miRNAs-mRNAs network and mutation signatures for finding potential molecular mechanisms underlying the development of LIHC. The biomarkers also can be used for predicting prognosis of LIHC.

## Introduction

Liver hepatocellular carcinoma (LIHC) has been one of the major causes of cancer mortality worldwide, which is the second highest cancer-related death disease in the world. There are 841,000 new cases of liver cancer and 782,000 deaths reports each year worldwide [1, 2]. Nowadays, the improvement of modern medicine treatment and combination of various therapeutic strategies can prolong the LIHC patient’s survival [3], however the outcome is still poor [4, 5]. Therefore, discovery of new means to diagnosis and prognosis is urgent and important.

Next generation sequencing is a breakthrough technology, which is redefining the landscape of human molecular genetic testing. Bioinformatics is a novelty cross discipline which develops and utilizes modern computational tools to analyze and interpret high dimensional biological data [6]. With the advances of next-generation sequencing technology, numerous disease-related genetic alterations have been revealed, bioinformatics has become an important component in clinical disease research. Progress in cancer genomics research over the past few decades has demonstrated that cancer is driven by various types of genomic alterations [7]. It has been reported that many types of tumourigenesis and development are closely associated with genomic alterations, such as papillary thyroid carcinomas [8], lung cancer [9], liver cancer [10], and etc.

Biomarker is designated as a parameter that can objectively be measured and evaluated as an indicator of normal biological processes, pathologic processes, or pharmacological response to therapeutic intervention [11]. Finding cancer biomarkers is available for early cancer detection, monitoring strategies, and tumor classification; so that the patient can receive the most appropriate therapy and that doctor can monitor the disease progression, regression, and recurrence [12]. Therefore, we urgently need to better understand the pathogenesis and progression of LIHC and to find specific biomarkers for diagnosis and prognosis.

Non-coding RNAs, such as micro RNAs (miRNAs) and long non-coding RNAs (lncRNAs), are defined as gene transcripts with little or no evident protein coding potential [13]. LncRNAs transcripts more than 200 nucleotides in length which are distinguished from other small ncRNAs such as tRNA, miRNA etc [14]. They are cell or tissue-specific in development processes or different disease. More and more evidence suggests that gene expression is regulated by lncRNAs at the transcriptional, post-transcriptional, and epigenetic levels [15]. Recently, it has increasing evidences indicated that many lncRNAs are involved in regulating tumorigenesis and progression via different biological processes, such as cell apoptosis [16], proliferation [17], and metastasis [18]. Those pathways in tumors are also regulated by lncRNAs and related to cancer diagnosis, prognosis, staging, and treatment [19–21].

MicroRNAs are small noncoding RNA molecule (about 22 nucleotides) that, by binding to complementary target mRNAs, can regulate cell proliferation, differentiation, and apoptosis [22]. The aberrantly expressed miRNA are found in many cancers [23], including lung cancer [24], neuroblastoma [25], and also liver cancer [26]. And it has diagnostic value for cancer diagnosis and therapy [27, 28]. There are many papers analyzed the differential expression and regulation of lncRNAs and miRNAs separately in LIHC [29, 30]. However, few researches have been studied on ceRNA of LIHC. Competitive endogenous RNAs (ceRNAs) are transcripts that can cross-regulate their ability by competing for shared miRNAs, thereby depredating or suppressing all target genes of the respective miRNA [31]. CeRNA networks link the function of mRNAs with microRNA, long non-coding RNA, and so on. A lot of ceRNAs have been reported successively, but few studies characterized such modulators of miRNA activity in LIHC [32].

The genome-scale screening analysis of LIHC was conducted to examine the relationship between the gene mutation signatures and its correlation with immune response. Several studies have shown that TP53 and CTNNB1 mutations tend to have a negative correlation with tumor immunity and immunotherapy response [33–35]. Besides TP53 and CTNNB1 mutations demonstrated a poor prognosis compared with wildtype. Titin (TTN) is a gene encoding a large abundant protein of striated muscle, which is divided into two regions, a N-terminal I-band and a C-terminal A-band. TTN mutation is frequently detected in LIHC, is associated with increased TMB and correlated with objective responsiveness to checkpoint blockades [36]. To detect the enrichment levels of immune signatures, we analyzed the LIHC cohorts from TCGA project and find the closed relationship between TTN mutation and immune infiltration.

In our study, LIHC samples from The Cancer Genome Atlas (TCGA) were obtained to screen differentially expressed genes (DEGs) between tumor and normal samples. We established a ceRNA-net signature. Univariable survival analyses were performed to identify prognostic genes of LIHC based on the intersection differentially expressed genes, miRNAs, and lncRNAs, mutation and clinical data.

## Materials and methods

### LIHC data sets collection

**Fig 1:**
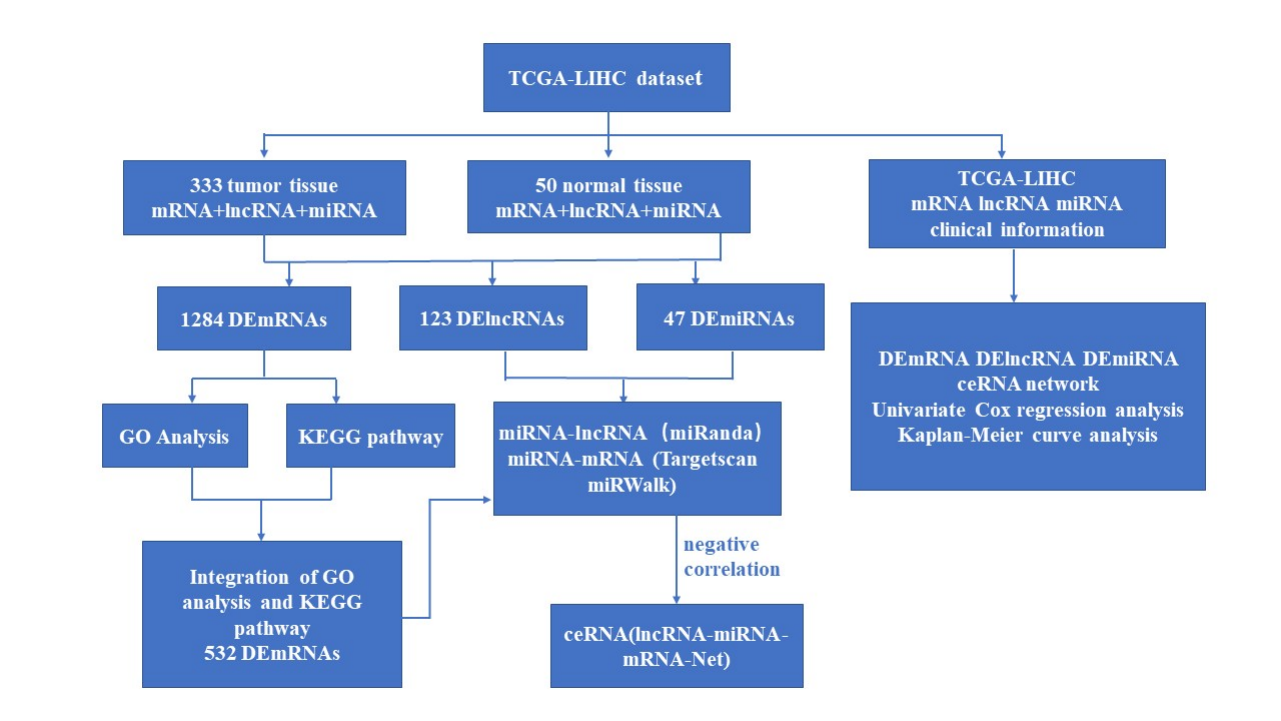
Flowchart of bioinformatics analysis for LIHC data from TCGA databases.

All the gene expression data (RNA-seqv2 & miRNA-seq) and corresponding clinical information were collected from The Cancer Genome Atlas (TCGA) database (https://portal.gdc.cancer.gov/). The screening criteria were as the following: (1) Studies with Liver Hepatocellular Carcinoma (LIHC) tissue samples; (2) the selected datasets containing lncRNA, mRNA and miRNA. (3) patients complicated without other tumors. Based on these criteria, a total of 333 tumor samples and 50 corresponding adjacent peritumoral samples were included. To investigate the lncRNA-miRNA-mRNA network associated with the pathogenesis and prognosis of LIHC and to identify novel molecular subtypes as potential biomarkers of LIHC. The LIHC data sets downloaded from the TCGA databases were integrated and analyzed by bioinformatics as the following workflow in Fig 1.

### Data processing and identification of differential expression genes

To integrated dysregulated lncRNAs, mRNAs and miRNAs across samples, hierarchical clustering was performed. Firstly, we used R statistical software (http://www.rproject.org/) to correct, normalize and calculate the raw data expression of RNA-seqv2 & miRNA-seq data. Next, differential expression of lncRNAs, mRNAs and miRNAs between tumor tissues and adjacent peritumoral tissues were identified through limma R package, and P < 0.05 & false discovery rate (FDR) < 0.05 & |log2FC|> 2 were set as statistically significant criterion. Total of 1284 mRNAs, 123 lncRNAs, 47 miRNAs were selected out for next step analysis.

### Functional enrichment analysis of differentially expressed mRNAs

To identify the meaningful functional annotation and pathway terms of differentially expressed mRNAs (DEmRNAs), we performed Gene Ontology (GO) classification and Kyoto Encyclopedia of Genes and Genomes (KEGG) pathway analysis among 1284 DEmRNAs using the online Bioinformatics Tool WebGestalt (http://www.webgestalt.org/) and KOBAS 3.0 (http://kobas.cbi.pku.edu.cn/index.php), respectively. Fisher test and Least Significant Difference-test were used to calculate the p value and False Discovery Rate. On the basis of test results, top 25 upregulated and downregulated GO and KEGG notable terms were generated. Then we identified the intersection genes of differentially expressed mRNAs included in both GO and KEGG pathways.

### Construction of ceRNA network

LncRNAs as a critical class of regulatory RNAs modulated the expression of mRNAs through competitively sponging miRNAs. miRNAs targeting bind both lncRNAs and mRNAs through specific base sequence pairing. From the result set obtained in step 2, we identified 123 DElncRNAs and 47 DEmiRNAs. Integration of GO analysis and KEGG pathway in step 6 made up the 532 DEmRNAs. In order to construct the competing endogenous regulating network, the miRanda (http://www.microrna.org/) was applied to match DEmiRNAs-DElncRNAs pairs. Meanwhile, the miRanda (http://www.microrna.org/), Targetscan (http://www.targetscan.org/), and miRWalk (http://129.206.7.150/) were used to predict the potential interaction of DEmiRNAs and DEmRNAs. According to miRNA negatively regulating target theory and Pearson correlation coefficients, we screened inverse relationship of DEmiRNA-DEmRNA, DEmiRNA-DElncRNA pairs and constructed the ceRNA network (lncRNA-miRNA-mRNA-network). Finally, we used the Cytoscape software to make the ceRNA network visualization.

### Protein-protein interaction network construction

To elucidate the underlying interaction of DEmRNAs involved in above ceRNA network, protein-protein interaction network was constructed using the STRING database (http://string-db.org/cgi/input.pl).

### Survival analysis

To determine the prognostic value of differential expression genes involved in ceRNA network in LIHC patients, we selected the data containing complete clinical information to perform the Kaplan‐Meier analysis using R software survival package. Firstly, the patients were divided into high expression level and low expression level two groups according to median expression of the differentially expressed gene including lncRNA, miRNA and mRNA. Then all the DERNAs were subjected to Univariate Cox regression analysis and survival curves were further calculated. p < 0.05 was considered as statistically significant.

### Mutation signatures and immune infiltrates analysis

We downloaded the Mutation annotation files (MAFs) from the GDC Data Portal (https://portal.gdc.cancer.gov), analyzed the mutation signatures containing top 30 mutated genes in LIHC. Among them, TTN ranking second only to TP53, demonstrated very high mutation rates. In addition, we explored the immune cell infiltration differences between TTN mutation and TTN wildtype, evaluated the correlation of OS and TTN mutation.

### Statistical analysis

Statistical analysis was performed using R (version 3.5.1, Auckland, NZ). The statistical analysis of categorical data was conducted with Fisher test and Least Significant Difference-test. The differences between two groups were assessed using “Deseq2” package with |log2 (fold change)| > 1 and FDR < 0.05. The Univariate Cox regression analysis was used for Kaplan‐Meier analysis of patient. p < 0.05 was considered to be statistically significant.

## Results

### Differential expression profile of RNAs in LIHC

**Fig 2:**
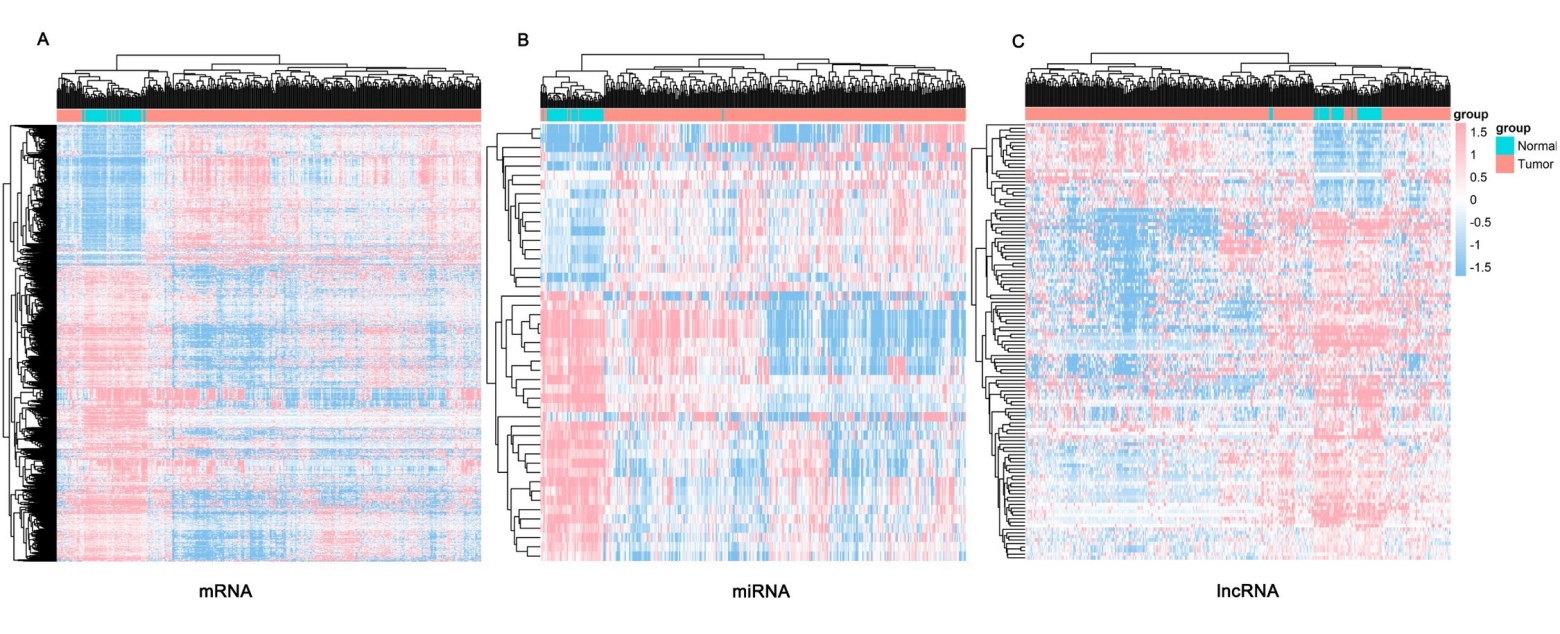
Differential expression profile of RNAs in LIHC. (A) Heat map of differentially expressed mRNAs. (B) Heat map of differentially expressed lncRNAs. (C) Heat map of differentially expressed miRNAs. The red represented the higher expression levels and the green represented lower expression levels. The rows and the columns represented the DERNAs and samples respectively.

To study the Differential expression profile of RNAs in LIHC, we corrected and normalized the RNA-seqv2 & miRNA-seq data using R statistical software (http://www.rproject.org/) before differential expression analysis. Then we performed unsupervised hierarchical clustering analysis to compare the difference expression RNAs between LIHC tumor tissues and adjacent normal tissues. A total of 123 differentially expressed lncRNAs (24 up-regulated and 99 down-regulated), 47 differentially expressed miRNAs (19 up-regulated and 28 down-regulated) and 1284 differentially expressed mRNAs (412 up-regulated and 872 down-regulated) were identified in RNA-seqv2 & miRNA-seq (p < 0.05 & |log2FC|> 2). DERNAs were visualized in heatmap (Fig 2).

### Functional enrichment analysis of the differentially expressed mRNAs

**Fig 3:**
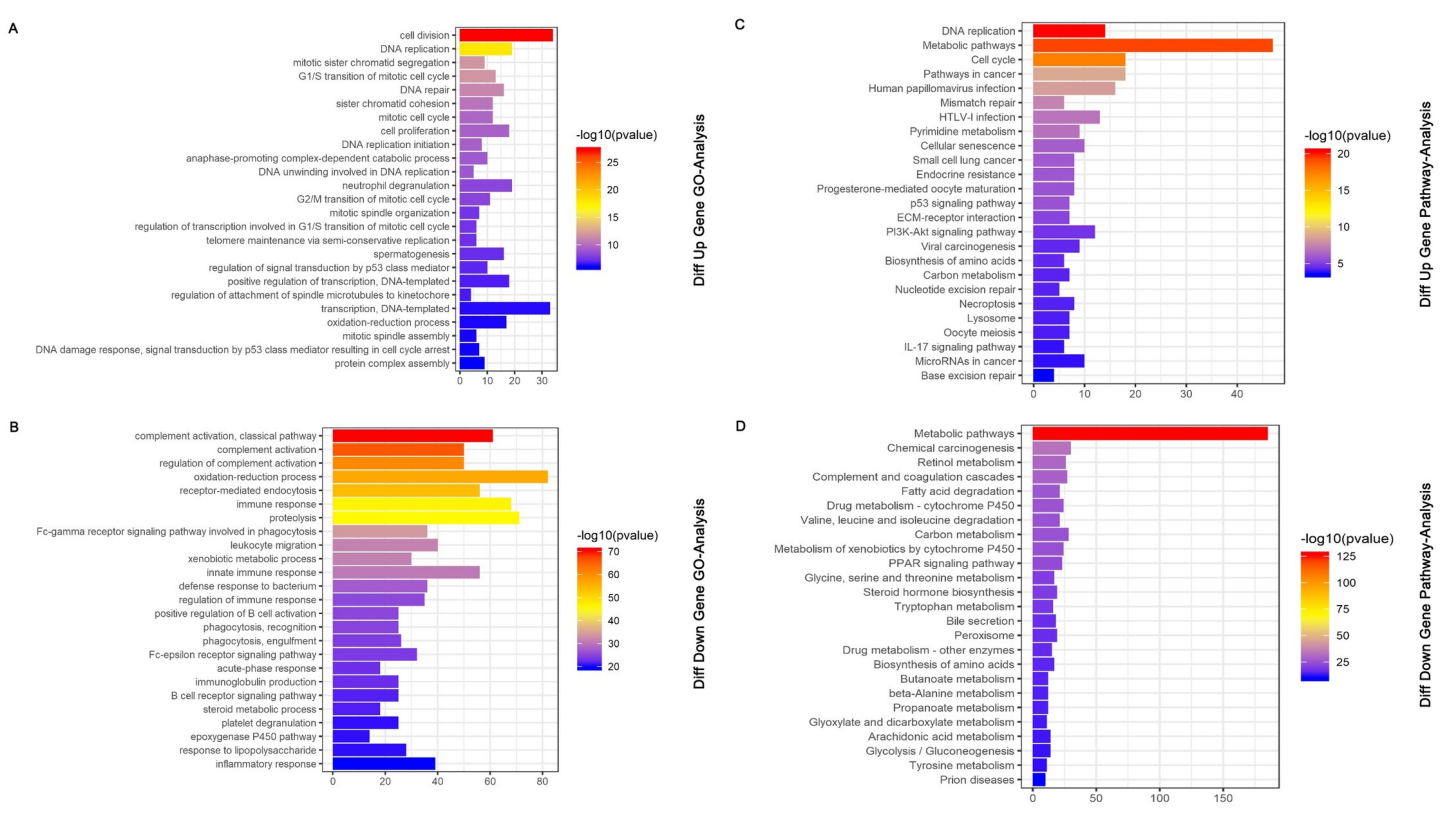
Functional enrichment analysis of the differentially expressed mRNAs. (A) Top 25 GO analyses of upregulated DEmRNAs. (B) Top 25 GO analyses of downregulated DEmRNAs. (C) Top 25 GO KEGG pathways of upregulated DEmRNAs. (D) Top 25 GO KEGG pathways of downregulated DEmRNAs.

To illustrates the meaningful functional pattern of differentially expressed mRNAs (DEmRNAs), a total of 1284 DEmRNAs were subjected to GO annotation and KEGG pathway analysis including 412 upregulated genes and 872 downregulated genes. We showed the top 25 GO analyses results (Fig 3A and 3B). GO analysis indicated that upregulated DEmRNAs were mainly associated with cell division, DNA replication, mitotic sister chromatid segregation. In contrast, downregulated DEmRNAs were mainly involved in complement activation, classical pathway, regulation of complement activation. The top 25 KEGG pathway terms were shown in Fig 3C and 3D. KEGG pathway terms revealed that upregulated DEmRNAs were primarily enriched in DNA replication, Metabolic pathways, cell cycle. While downregulated DEmRNAs were primarily involved in Metabolic pathways, chemical carcinogenesis, retinol metabolism.

### CeRNA network

**Fig 4:**
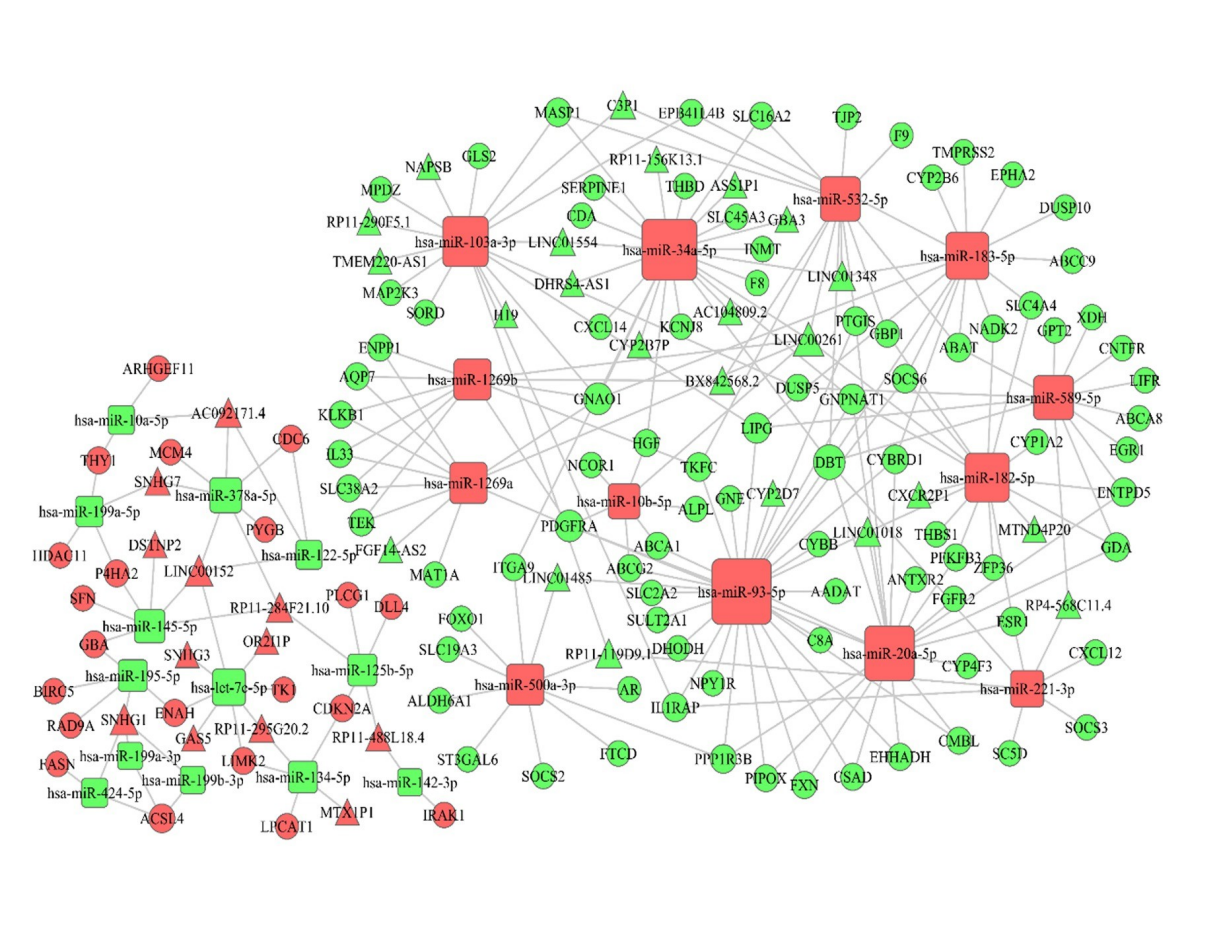
LncRNA-miRNA-mRNA network of DERNAs. Red stands for upregulated genes and green stands for downregulated genes. circles represent mRNAs, quadrates represent miRNAs, diamonds represent lncRNAs. The point size corresponds to regulatory ability of genes. Larger point means stronger regulatory ability.

LncRNAs modulated the expression of mRNAs through competitively sponging miRNAs. miRNAs bind to the 3′untranslated region (3′- UTR) of lncRNAs and mRNAs through specific base sequence pairing leading to RNAs degradation. According to miRNA negatively regulating target theory and co-expression relationship, first we identified 123 DElncRNAs, 47 DEmiRNAs from step 2 and 532 DEmRNAs from step 6. Next, we predicted the target binding of mRNAs, lncRNAs with miRNAs using miRanda, Targetscan and miRWalk websites. Then we screened inverse relationship of DEmiRNA-DEmRNA, DEmiRNA-DElncRNA pairs and constructed the ceRNA network (lncRNA-miRNA-mRNA-network) using Cytoscape software. As a result, 542 lncRNAs-miRNAs-mRNAs pairs were predicted to construct a ceRNA regulatory network including 35 DElncRNAs, 26 DEmiRNAs and 112 DEmRNAs. Among them, has-miR-93-5p, has-miR-34a-5p, has-miR-20a-5p, has-miR-103a-3p, has-miR-183a-5p, has-miR-182a-5p displayed stronger targeting function than other miRNAs, which indicated that they had important functions in regulating other genes (Fig 4).

### Protein-Protein Interaction (PPI) network construction

**Fig 5:**
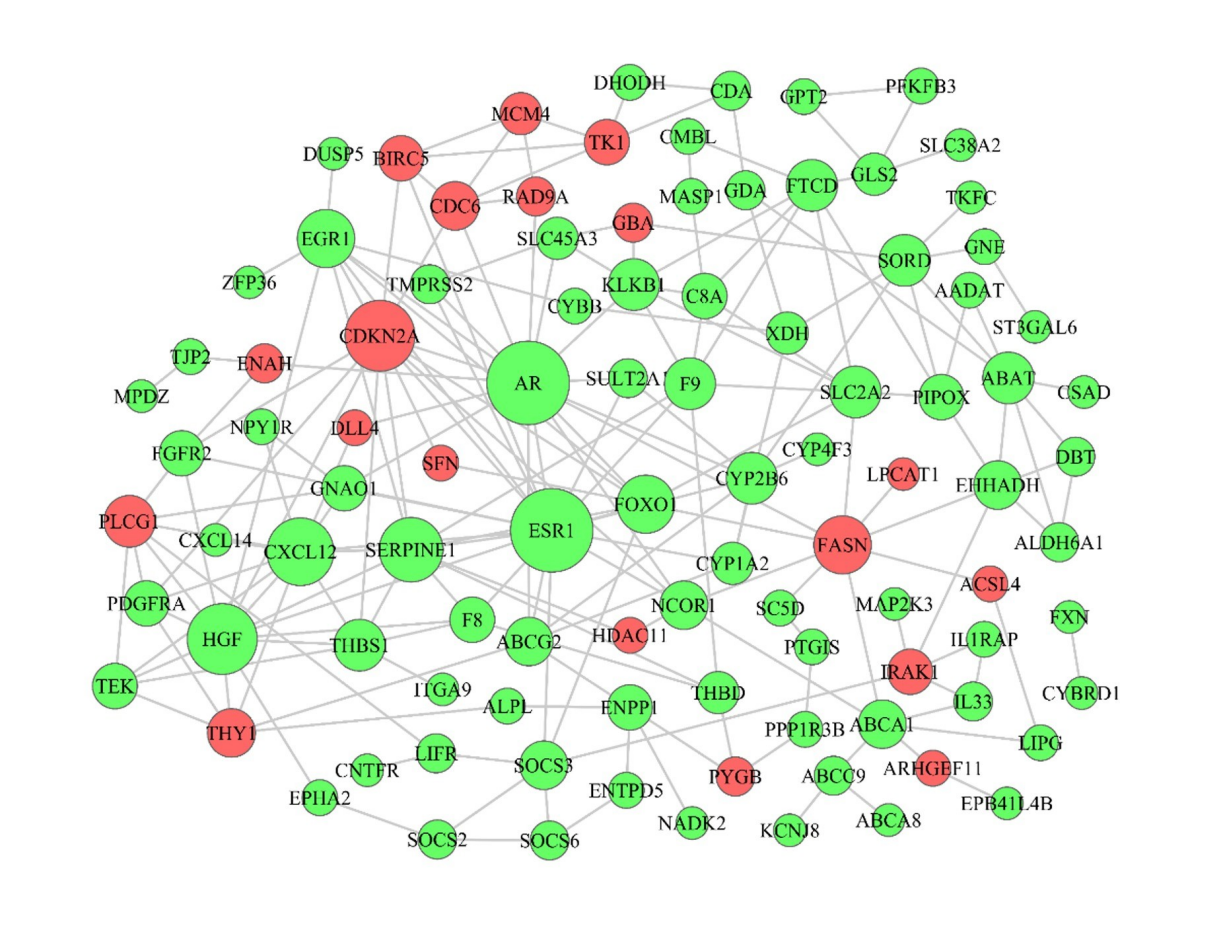
Protein–protein interaction network. All the points are differentially expressed mRNAs, node size represents regulatory capacity of mRNAs. The regulatory capacity was stronger with larger size. Green represents downregulated mRNAs. Red represents upregulated mRNAs.

Based on ceRNA network, we get 112 differentially expressed mRNAs including 21 upregulated mRNAs and 91 downregulated mRNAs. To elucidate the underlying interaction of DEmRNAs involved in above ceRNA network, mRNAs with interconnected relationships were selected to construct the protein-protein interaction network using the STRING website. The PPI network was composed of links and nodes, whose size represented the regulatory capacity of mRNAs. Among them, the upregulated gene CDKN2A and downregulated genes ESR1, AR had the closest connection to other proteins (Fig 5).

### Kaplan-Meier curve analysis of DERNAs

**Fig 6:**
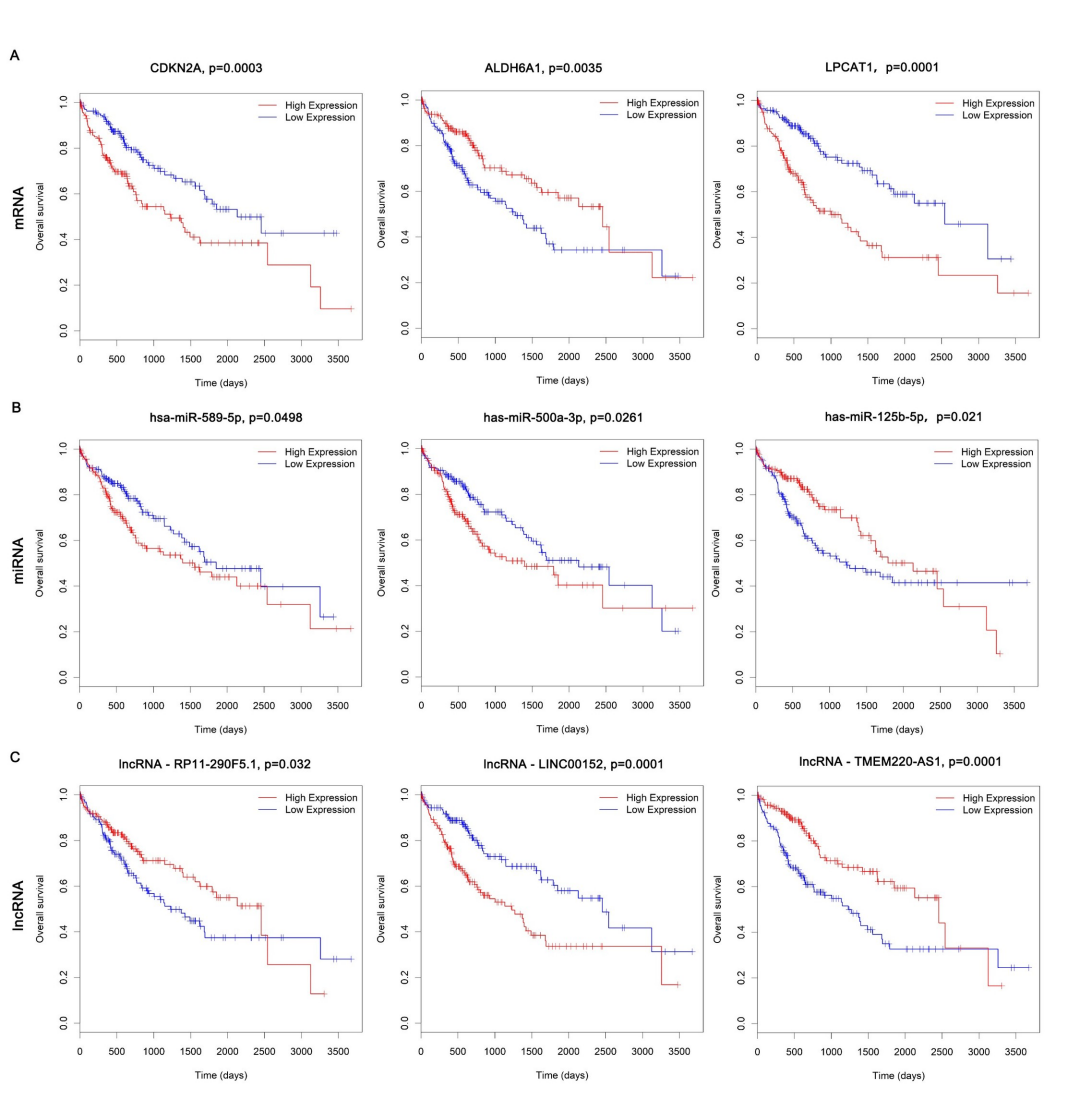
Kaplan-Meier curves of the 12 DERNAs associated with overall survival. (A) DEmRNAs associated with OS, (B) DEmiRNAs associated with OS, and (C) DElncRNAs associated with OS.

Liver hepatocellular carcinoma (LIHC) is the second leading cause of cancer death in the world. Prolonging overall survival is considered as the ultimate goal of LIHC patients. It is very important to comprehensively investigate the utility of lncRNAs, mRNAs and miRNAs in LIHC as potential biomarkers for prognosis. Therefore we selected the data containing complete clinical information to perform the Kaplan‐Meier analysis using R software survival package. Then, DERNAs involved in ceRNA network were subjected to Univariate Cox regression analysis and survival curves were further calculated. Total of 43 mRNAs, 14 lncRNAs and 3 miRNAs were screened out to be significantly correlated with overall survival (OS) of LIHC. Among the DEmRNAs, the upregulated gene of LPCAT1 and CDCKN2A were the most significantly negatively correlated with OS (p = 0.0001 and p = 0.0003 respectively), the downregulated gene of ALDH6A1 was the most significantly positively correlated with OS (p = 0.0035). For DEmiRNAs, the upregulated has-miR-589-5p and has-miR-500a-3p most significantly suggested a shorter survival (p = 0.0498 and p = 0.0261 respectively). The downregulated has-miR-125b-5p most significantly suggested a longer survival (p = 0.021). A low expression of LINC00152 linked most significantly to the longer OS (p = 0.0001). Meanwhile, the high expression of RP11-290F5.1 and TMEM220-AS1 linked significantly to the shorter OS (p = 0.032 and p = 0.0001 respectively). Survival curves results included lncRNAs, mRNAs and miRNAs (Fig 6). Cox regression analysis was used to identify prognostic ceRNAs (Table 1). We verified the expression of prognostic ceRNAs in our own samples, which was consistent with the results of TCGA (S1 Fig).

**Table 1:** Identify the biomarkers to predict prognosis based on multiple variate COX model.

Characteristics	Univariate cox			Multivariate cox		
	Hazard.Ratio	CI95	P. Value	Hazard.Ratio	CI95	P. Value
ALDH6A1	0.58	0.4–0.84	0.00394	0.68	0.44–1.04	0.077
AR	0.64	0.44–0.93	0.01834	0.92	0.6–1.43	0.723
FTCD	0.63	0.44–0.92	0.01549	0.64	0.43–0.94	0.024
hsa.miR.500a.3p	1.52	1.05–2.19	0.02717	1.44	0.98–2.1	0.061
RP11.119D9.1	0.64	0.44–0.93	0.01785	0.92	0.59–1.43	0.709
SOCS2	0.47	0.32–0.69	0.00012	0.51	0.34–0.76	0.001

### Comparisons of the mutation signatures and its correlation with immune infiltrates

**Fig 7:**
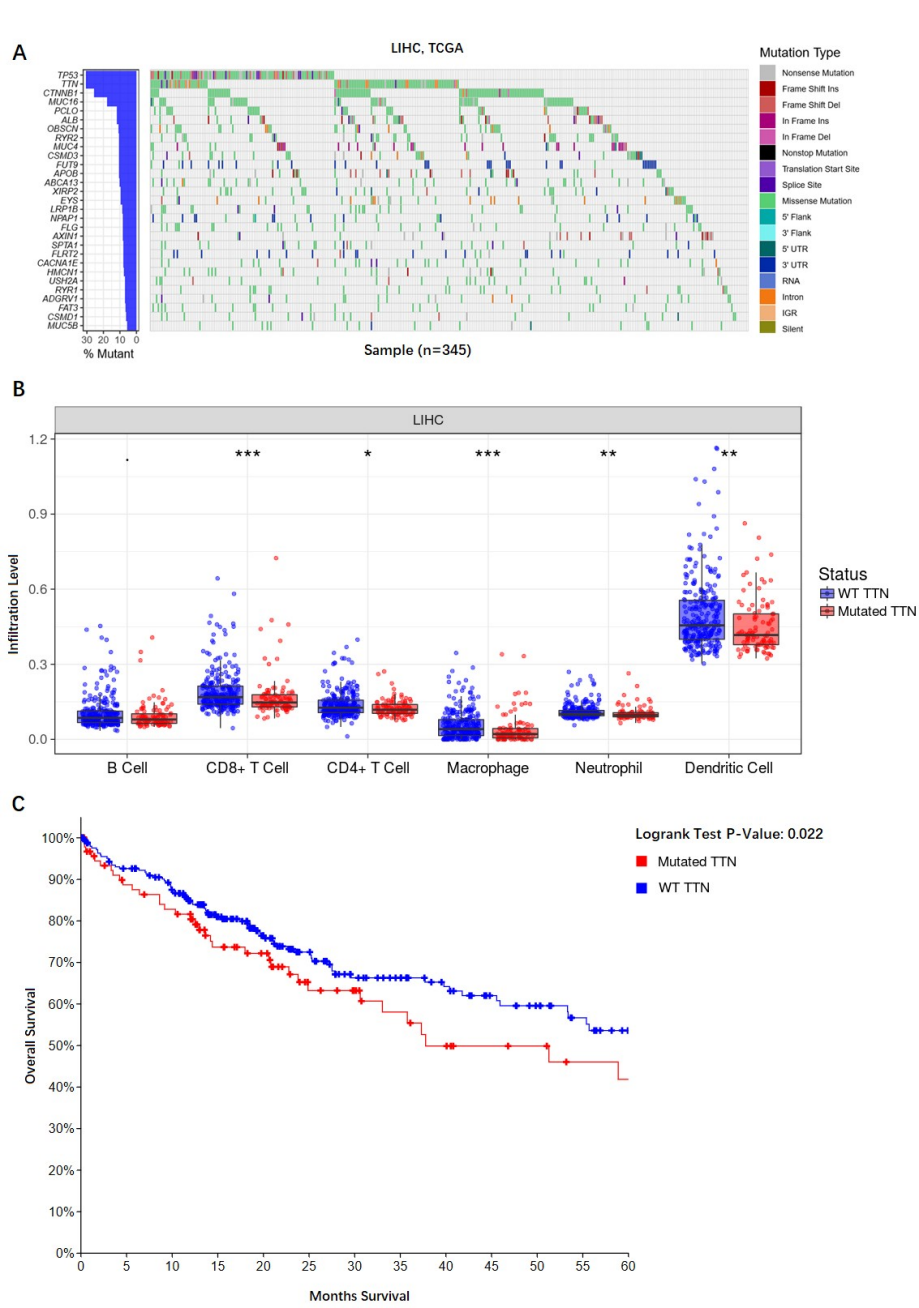
Identification of mutation signatures and its correlation with the immune infiltration and overall survival. (A) Heatmap representation of the distribution of driver genes mutations in patients, (B) The immune signatures (CD8^+^ T cells, Macrophage, Neutrophil, and Dendritic Cells) show significantly higher enrichment levels in TTN- wildtype than in TTN- mutated cancers in LIHC, (C) Kaplan-Meyer survival curves showed that TTN mutation have a negative correlation with OS in LIHC.

We analyzed the characteristic pattern of mutated genes of 345 LIHC from TCGA, among them, TP53, TTN, CTNNB1, MUC16 and PCLO had the highest mutation frequency (Fig 7A). We further compared the immune signatures (B cell, CD8^+^ T cell, CD4^+^ T cell, Macrophage, Neutrophil and Dendritic cell) between mutated and wildtype genes in LIHC. we found that the immune signatures (CD8^+^ T cells, CD4^+^ T cell, Macrophage, Neutrophil and Dendritic cell) showed significantly higher enrichment levels in TTN-wildtype than in TTN- mutated cancers in LIHC (P < 0.01) (Fig 7B). Previous studies have indicated that TP53 mutations play a negative role in antitumor immunity. However, immune infiltrates showed no significantly difference between TP53-mutated and TP53-wildtype in LIHC (P < 0.01). Then, we examined the correlation between TTN mutations and five years OS, we found that TTN mutation weas significantly associated with a worse OS in LIHC (Fig 7C).

## Discussion

LIHC is the second highest cancer-related death in the world, with a steady rise of the incidence every year [37]. Nowadays, surgical resection, ablation by radiofrequency or ethanol injection, and liver transplantation are used on early stage LIHC patients, however the overall five-year survival rate still ranges between 50% and 70% [38]. Therefore, there is an urgent need to find genes that associated with the pathogenesis and progression in LIHC and to search biomarkers for diagnosis and prognosis. Many studies have reported that LIHC patients have certain dysregulated expression of mRNAs [2], miRNAs [39], and lncRNAs [40]. However, there are few studies about the crosstalk between mRNAs, miRNAs, and lncRNAs. Single-cell transcriptome-based multilayer network biomarker for predicting prognosis and therapeutic response of gliomas [41]. Recently, ceRNAs were be identified that play important roles in the physiology and development of cancer [32]. And ceRNA interactions are different in different kind of cancers [42]. Systematic studies that evaluate ceRNAs in LIHC are need.

In this study, mRNA, miRNA, and lncRNA data from TCGA were used to identify the differential expression profiles. Gene oncology analysis revealed that the upregulated genes were primarily enriched in cell division, DNA replication, and mitotic sister chromatid segregation. The downregulated genes were primarily involved in complement activation, classical pathway, complement activation, regulation of complement activation. According to the results from KEGG pathway analysis, upregulated genes play an important role in DNA replication, metabolic pathways, and cell cycle. The top three down-regulated gene enrichment pathways are metabolic pathways, chemical carcinogenesis, and retinol metabolism. We constructed a ceRNA interplay networks to search out RNAs that regulates each other. Based on the mRNA in this regulatory network, PPI network analysis was constructed to annotate the correlations between the differentially expressed genes, and we found that ESR1, AR, HGF, CDKN2A, and CXCL12 are essential in regulatory networks. We are recommitted to pursuing the biomarkers that more accurately predict the prognosis of LIHC to improve personalized cancer therapy. For this purpose, we analysed the clinical data and possibilities of using those RNAs as diagnostic biomarkers or therapeutic targets.

Many ceRNAs have been found to play vital roles in tumour progression. The abundance of other RNA transcripts can be altered by ceRNAs. In order to understand the biological implications of the miRNAs which were involved in the ceRNA network, we conducted lncRNA-miRNA-mRNA network analyses and identified 25 ceRNAs, including up-regulated miR-103a-3p, miR-1269b, miR-1269a, miR-34a-5p, miR-532-5p, miR-93-5p, miR-500a-3p, miR-20a-5p, miR-183-5p, miR-589-5p, miR-182-5p, miR-221-3p, and down-regulated miR-10a-5p, miR-378a-5p, miR-199a-5p, miR-122-5p, miR-145-5p, miR-195-5p, miR-125b-5p, miR199a-3p, miR199b-3p, miR-424-5p, miR-134-5p, miR-142-3p. To further determine the correlations between the differentially expressed genes in ceRNA network and survival, we used Kaplan-Meier survival analysis to evaluate the prediction prognostic biomarkers of LIHC. We found 3 miRNAs that are strongly related to prognosis, including miR-589-5p, miR-500a-3p, and miR-125b-5p. There have been some studies on these miRNAs in tumour-associated research. The overexpressed miR-589-5p maintains the stemness of hepatocellular carcinoma cells and promotes chemoresistance [43]. However, in another study, miR-589-5p was found to inhibit the stemness of LIHC cancer stem cells (CSCs) through MAP3K8 and miR-589-5p down-regulation in LIHC is associated with a poor clinical prognosis [44]. We found that miR-589-5p is overexpressed in LIHC and associated with a poor clinical prognosis. MiR-500a-3p was shown to promote the stemness maintenance of CSCs via JAK/STAT3 signaling pathway in LIHC [45]. MiR-125b-5p functions as an oncogene and a prognostic biomarker in LIHC [46]. All the studies on miRNA in LIHC demonstrated the importance of those miRNAs, but the regulatory relationships between these miRNAs and mRNAs or lncRNAs are still unclear. This is the first report that includes annotated ceRNAs in LIHC, which may provide new therapeutic possibilities for patients.

Genomic alterations are hallmarks of many types of cancers, the same to LIHC. We detected 43 mRNAs that are obviously related to prognosis, including CDKN2A, ALDH6A1, LPCAT1, and so on. Although these genes perform well in LIHC prognosis prediction, due to the limitation of the samples in the datasets, larger cohorts and experimental validation are needed. We use the TIMER database to explore the relationship between TTN mutation and immune infiltration in LIHC. We find the negative relationship of TTN mutation and immune cells infiltration including CD8^+^ T cell, CD4^+^ T cell, Macrophage, Neutrophil and Dendritic cell. In addition, our survival analysis revealed TTN mutation predicts a poor prognosis. Thus we infer that TTN plays a pivotal role in the regulation and recruitment of immune cell infiltration in LIHC and can serve as a therapeutic target.

Overall, lncRNA-miRNA-mRNA network analyses were performed for prediction of important ceRNAs in LIHC. Cox proportional hazards regression analysis was used to identify significant prognostic differentially expressed genes. Immune infiltration analysis showed the importance of mutation signature in tumor immunity. between mutation These findings could advance the accuracy of diagnosis and prognosis and might aid in the development of targeted therapy for LIHC.

## Supporting information

S1 Fig We verified the expression of prognostic ceRNAs in our own sample. (DOCX)
