# Supplementary figures and images for: Construction of liver hepatocellular carcinoma-specific lncRNA-miRNA-mRNA network based on bioinformatics analysis
Source: PLoS One. 2021 Apr 16;16(4):e0249881. doi: 10.1371/journal.pone.0249881 (PMC8051809; doi:10.1371/journal.pone.0249881)

S1 Fig: We verified the expression of prognostic ceRNAs in our own sample.
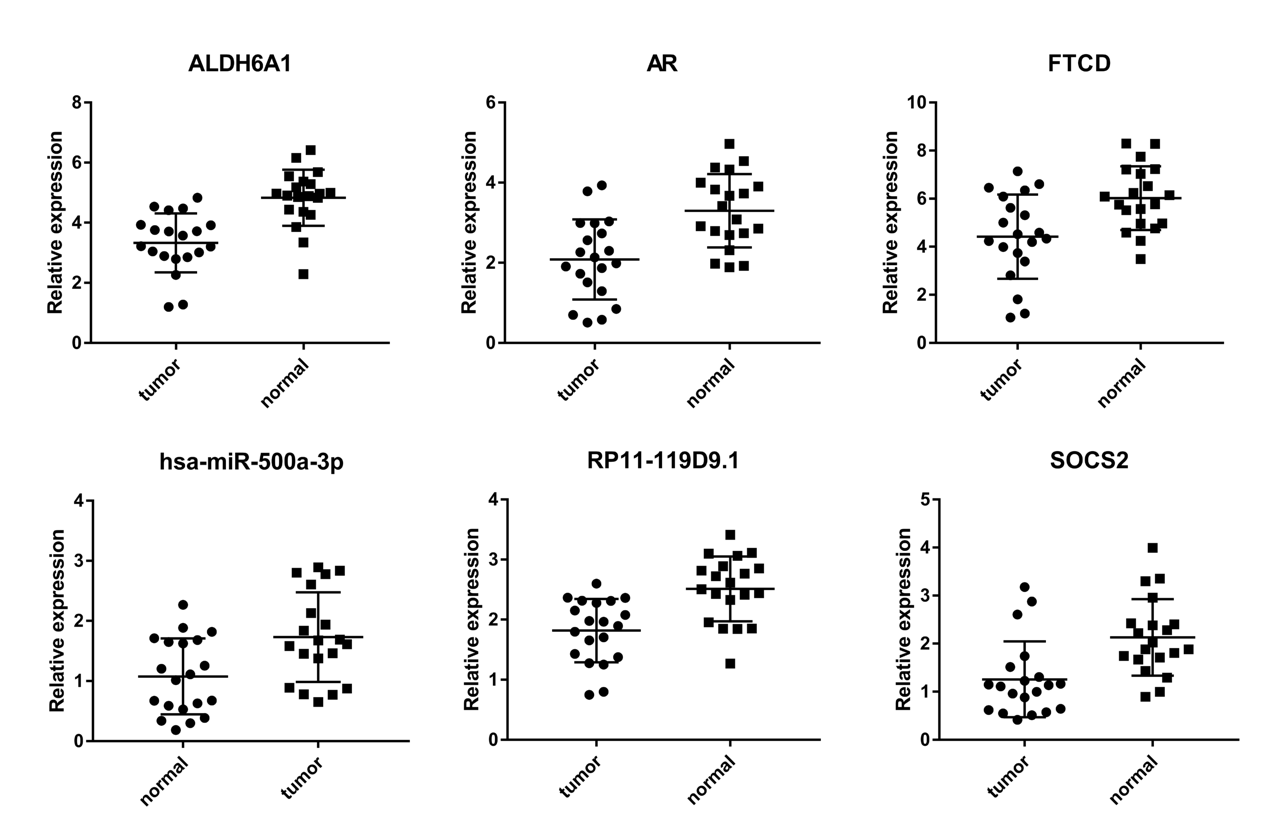

Supplement: S1 Fig — (DOCX) [file pone.0249881.s001.docx]
